# Drowning the kidneys: from fluid overload to physiology-guided fluid management in onco-hematology

**DOI:** 10.1093/ckj/sfag232

**Published:** 2026-07-13

**Authors:** Fernando Parra-Londoño, Marina Urrutia-Jou, Jordi Soler-Majoral, Jordi Ara-Bonet, Desirée Catalán, Marta García, Mohamed Nassiri-Nassiri, Cristhian Paul Cedeño-Parra, Daniela Buccione, Néstor Rodríguez-Chitiva, Susana Vives, Carolina Tudela, Javier A Neyra, Faeq Husain-Syed, María José Soler, Gregorio Romero-González

**Affiliations:** Nephrology Department, Germans Trias i Pujol University Hospital, Badalona, Spain; Nephrology Department, Germans Trias i Pujol University Hospital, Badalona, Spain; REMAR-IGTP Group, Germans Trias i Pujol Research Institute, Can Ruti Campus, Badalona, Spain; Nephrology Department, Germans Trias i Pujol University Hospital, Badalona, Spain; REMAR-IGTP Group, Germans Trias i Pujol Research Institute, Can Ruti Campus, Badalona, Spain; Cardiology Department, Germans Trias i Pujol University Hospital, Badalona, Spain; Nephrology Department, Germans Trias i Pujol University Hospital, Badalona, Spain; Nephrology Department, Germans Trias i Pujol University Hospital, Badalona, Spain; Nephrology Department, Germans Trias i Pujol University Hospital, Badalona, Spain; Hematology Department, ICO Badalona – Germans Trias i Pujol University Hospital, Institut de Recerca Josep Carreras, Badalona, Spain; Unitat Hospitalització Oncològica i Unitat d’Atenció Continuada Oncològica, Institut Català d’Oncologia, Badalona, Spain; Nephrology Department, Germans Trias i Pujol University Hospital, Badalona, Spain; REMAR-IGTP Group, Germans Trias i Pujol Research Institute, Can Ruti Campus, Badalona, Spain; Hematology Department, ICO Badalona – Germans Trias i Pujol University Hospital, Institut de Recerca Josep Carreras, Badalona, Spain; Unitat Hospitalització Oncològica i Unitat d’Atenció Continuada Oncològica, Institut Català d’Oncologia, Badalona, Spain; Department of Medicine, Division of Nephrology, University of Alabama at Birmingham, Birmingham, AL, 35294, USA; Department of Internal Medicine II, University Hospital Giessen and Marburg, Justus-Liebig-University Giessen, Giessen, Germany; Nephrology Department, Hospital Vall d`Hebron, Barcelona, Spain; Nephrology Department, Germans Trias i Pujol University Hospital, Badalona, Spain; REMAR-IGTP Group, Germans Trias i Pujol Research Institute, Can Ruti Campus, Badalona, Spain; International Renal Research Institute of Vicenza, Vicenza, Italy

**Keywords:** acute kidney injury, congestive nephropathy, fluid overload, hematologic malignancies, onco-nephrology

## Abstract

Acute kidney injury (AKI) is a frequent complication in patients with cancer. Although AKI in oncology is often multifactorial, it is frequently attributed to nephrotoxicity or intrinsic tubular injury. However, its hemodynamic mechanisms remain underrecognized. Accumulating evidence from critical care and cardiorenal medicine indicates that fluid overload, venous congestion, and increased intra-abdominal pressure are major drivers of kidney dysfunction and adverse outcomes in patients with cancer. These mechanisms are particularly relevant in onco-hematology, where protocol-driven hydration, transfusions, systemic inflammation, and capillary leak are common. This review examines venous congestion as an underrecognized and potentially reversible contributor to AKI in patients with cancer, by integrating epidemiological data, mechanistic insights, and clinical evidence. Particular emphasis is placed on the distinction between fluid responsiveness (FR) and fluid tolerance (FT), with the reviewed studies suggesting that many patients with AKI are neither fluid responsive nor fluid tolerant and that these phenotypes evolve dynamically over time. Tumor lysis syndrome is presented as a paradigmatic clinical scenario in which aggressive hydration is widely recommended despite limited high-quality evidence and a substantial risk of fluid intolerance. We further describe the role of point-of-care ultrasound in the bedside assessment of cardiac filling pressures, venous congestion, and stroke volume, and propose a physiology-based framework for AKI evaluation that prioritizes exclusion of urinary tract obstruction, systematic assessment of FT, and selective evaluation of FR in hypotensive or low-flow states. Adopting this approach may help individualize fluid management, reduce iatrogenic harm, and improve renal outcomes in patients with cancer.

## CASE REPORT

A 50-year-old woman with newly diagnosed NPM1-mutated, FLT3-high acute myeloid leukemia started induction chemotherapy with daunorubicin, cytarabine, and midostaurin. During induction, she received continuous maintenance intravenous fluids (∼2 l/day), parenteral nutrition, antimicrobial therapy, and multiple red blood cell and platelet transfusions. Three days after treatment initiation, she developed dyspnea and hypoxemia. Chest radiography showed bilateral basal infiltrates and pleural effusions. Initial management included intravenous furosemide for suspected fluid overload. On day 5 of treatment, nephrology was consulted because of acute kidney injury [serum creatinine increased from a baseline of 0.8 to 2.5 mg/dl; Kidney Disease: Improving Global Outcomes (KDIGO) stage 3]. The patient reported exertional dyspnea and orthopnea. Physical examination revealed bilateral pulmonary crackles. Point-of-care ultrasound (PoCUS) demonstrated diffuse bilateral B-lines, a moderate right pleural effusion, a small left pleural effusion, a dilated noncollapsible inferior vena cava (24 mm), and pulsatile portal vein flow, consistent with systemic and pulmonary congestion. Renal ultrasound showed no urinary tract obstruction. Cardiology assessment revealed preserved biventricular systolic function, moderate mitral and tricuspid regurgitation, grade II diastolic dysfunction, estimated pulmonary artery systolic pressure of 35 mmHg, and N-terminal pro-B-type natriuretic peptide (NT-proBNP) of 4328 pg/ml, supporting the diagnosis of congestive cardiorenal syndrome. Intravenous fluids were discontinued and intravenous diuretics intensified. Serial PoCUS examinations showed progressive improvement in pulmonary and venous congestion, with restoration of continuous portal and intrarenal venous flow patterns. Serum creatinine decreased to 1.1 mg/dl and body weight fell from 63 to 59.8 kg, supporting venous congestion as an important reversible contributor to kidney dysfunction.

## INTRODUCTION

Acute kidney injury (AKI) is a common and clinically significant complication in patients with cancer, affecting both hematologic and solid malignancies, particularly in those receiving systemic therapy. Data summarized by Yarandi *et al*. indicate that AKI occurs in 15%–30% of hospitalized cancer patients, with even higher rates in high-risk settings such as acute leukemias, aggressive lymphomas, and multiple myeloma (20%–50%), during intensive chemotherapy, and following hematopoietic stem cell transplantation (HSCT; 30%–70%) [1]. In patients receiving immune-based therapies, AKI has been reported in up to 15%–25% of cases, although directly immune-mediated kidney injury is less common (1%–5%).

AKI often represents a critical turning point in oncology and hematology care, as it can lead to treatment delays, dose reductions or discontinuation, increased need for supportive care, prolonged hospitalization, and worse overall outcomes. In a large population-based cohort of over 160 000 patients initiating systemic anticancer therapy, Kitchlu *et al*. reported that 9.3% experienced hospitalization for AKI or required acute dialysis. The burden was particularly high in multiple myeloma, leukemia, and bladder cancer, with cumulative incidences of 15%–26%, and the risk was more than doubled within the first 90 days after treatment initiation [2]. These findings highlight the early treatment phase as a period of heightened vulnerability.

Traditionally, AKI in oncology is considered multifactorial, including tumor-related injury, treatment-induced nephrotoxicity, sepsis, and obstruction. However, in clinical practice, it is often oversimplified as acute tubular injury, especially in the presence of chemotherapy, immunotherapy, or infection. This approach fails to capture the complex and overlapping mechanisms involved [3]. Increasing attention has been given to the role of fluid tolerance (FT), fluid overload (FO), and venous congestion (VC) in both the development and worsening of AKI. In this review, FO refers to excessive fluid accumulation associated with adverse physiological consequences, whereas VC specifically denotes elevated venous pressures contributing to organ dysfunction. Although related, these terms are not interchangeable and may reflect different physiological mechanisms. Evidence from critical care and cardiorenal medicine shows that FO, VC, and increased intra-abdominal pressure (IAP) are key contributors to kidney dysfunction and adverse outcomes [4–6]. Congestion-related kidney injury is frequently overlooked and should instead be viewed as a contributing mechanism rather than an alternative diagnosis [7].

Among tumor-related causes, tumor lysis syndrome (TLS) remains a classic and important driver of AKI, particularly in highly proliferative hematologic malignancies such as acute myeloid leukemia (AML). TLS occurs in 5%–70% of patients depending on disease type and treatment intensity and is associated with high morbidity and mortality [8–10]. Standard prophylaxis includes aggressive intravenous hydration (>2 l/m^2^/day) and allopurinol (Table 1). However, there is no randomized evidence demonstrating that aggressive hydration reduces AKI or improves outcomes. On the contrary, excessive fluid administration may increase the risk of FO, VC, and secondary organ dysfunction [10]. Among the potential consequences of this process is congestive nephropathy, a potentially reversible form of kidney injury caused by impaired renal venous outflow and increased interstitial pressure, often without overt tubular damage [5, 6]. This mechanism is especially pertinent in onco-hematology patients exposed to high fluid volumes, transfusions, corticosteroids, and systemic inflammation. Furthermore, recent frameworks distinguish fluid responsiveness (FR) from FT, emphasizing that increases in cardiac output (CO) may still occur alongside harmful congestion [11, 12].

**Table 1:** Fluid volume recommendations for TLS prophylaxis and related prevention strategies.

Reference	Clinical setting	Recommended fluid volume	Equivalent volume for a standard adult (70 kg, BSA 1.73 m^2^)	Additional remarks
Coiffier *et al*., 2008 [68]	Intermediate- and high-risk TLS; established laboratory TLS (LTLS)/clinical TLS (CTLS)	2–3 l/m^2^/day IV	3.5–5.2 l/day	One-quarter normal saline with 5% dextrose; potassium, calcium, and phosphate initially withheld from hydration fluids.
Jones *et al*. (BCSH, 2015) [69]	TLS prophylaxis in adults with hematological malignancies	Aim for 3 l/24 hour in adults	3.0 l/day	Exact fluid requirement is stated to be unknown; 3 l/day considered a reasonable target.
Chan *et al*. (BSH, 2025) [70]	TLS prophylaxis in adults with hematological malignancies	Oral fluid intake of 2–3 l/day starting the day before treatment	2–3 l/day	Intravenous hydration should be used if oral intake is inadequate, particularly in high-risk patients.
Fischer *et al*. (American Society of Hematology [ASH] Education Program, 2020) [71]	Venetoclax initiation in CLL (low TLS risk)	Oral hydration 1.5–2 l/day beginning 2 days before dose escalation	1.5–2 l/day	Combined with urate-lowering therapy and laboratory monitoring.

Volumes are reported as stated in the original guideline or publication. When applicable, equivalent adult volumes were calculated using a body surface area of 1.73 m^2^ solely to facilitate comparison across recommendations. Pediatric recommendations should not be extrapolated to adult practice.

BCSH, British Committee for Standards in Haematology; BSH, British Society for Haematology; BSA, body surface area; CLL, chronic lymphocytic leukemia; IV, intravenous.

Diagnosis of AKI typically relies on serum creatinine, urine output, and physical examination, which provide limited insight into underlying hemodynamics, especially in the presence of FO and congestion. These limitations have increased interest in bedside tools capable of providing physiological and hemodynamic assessment beyond conventional markers, particularly point-of-care ultrasound (PoCUS) [13–15].

This review addresses the contribution of VC and FO to AKI in onco-hematology, evaluates the impact of current hydration strategies, and explores the role of PoCUS in identifying and managing potentially reversible, congestion-related kidney injury.

## METHODS

A structured literature search was conducted in PubMed and Embase up to February 2026 using terms related to acute kidney injury, fluid overload, venous congestion, tumor lysis syndrome, and point-of-care ultrasound. Observational studies, clinical trials, systematic reviews, and position papers relevant to onco-nephrology, critical care, and cardiorenal medicine were considered. Given the limited oncology-specific evidence, selected data from related fields were included when considered clinically applicable. This review was guided by clinical relevance and aimed to provide a physiology-based framework rather than a systematic synthesis of evidence. No formal study selection process, risk-of-bias assessment, or language restrictions were applied.

### Fluid overload as an underrecognized driver of acute kidney injury in the oncology and hematology settings

Both Yarandi *et al*. and Kitchlu *et al*. identify well-established contributors to AKI, including pre-existing chronic kidney disease (CKD), diabetes, advanced cancer stage, sepsis, nephrotoxic agents, and postrenal obstruction [1, 2]. However, the etiology of AKI is frequently described in broad and nonspecific terms, often labeled as multifactorial or attributed to prerenal azotemia, without a systematic evaluation of fluid exposure, volume status, or congestion. In cancer patients, emerging evidence indicates that FO is not simply a consequence of organ dysfunction but may independently contribute to adverse outcomes. For example, in a prospective cohort of 122 critically ill cancer patients admitted to the intensive care unit (ICU), a positive fluid balance at admission was independently associated with increased ICU mortality, even after adjusting for disease severity. Overall ICU mortality was 15.5%, and each additional 100 ml of positive fluid balance over 24 hours increased the odds of death by 3% [16], demonstrating a clear dose–response relationship.

Beyond oncology-specific populations, critical care data consistently link FO with AKI. A synthesis by Babroudi *et al*. demonstrates that positive fluid balance is associated with increased risk of AKI, impaired renal recovery, greater need for renal replacement therapy (RRT), and higher mortality, even in the absence of hypotension [4]. These findings support the concept that FO may act as an upstream driver of kidney injury rather than merely reflecting disease severity. Similar observations have been reported in hematologic malignancies: in patients with AML undergoing induction therapy, FO occurred in ∼12% of cases and was independently associated with higher 90-day mortality. Increased crystalloid administration was identified as a major risk factor for FO [17].

#### Pathophysiology of congestion-mediated AKI

**Figure 1:**
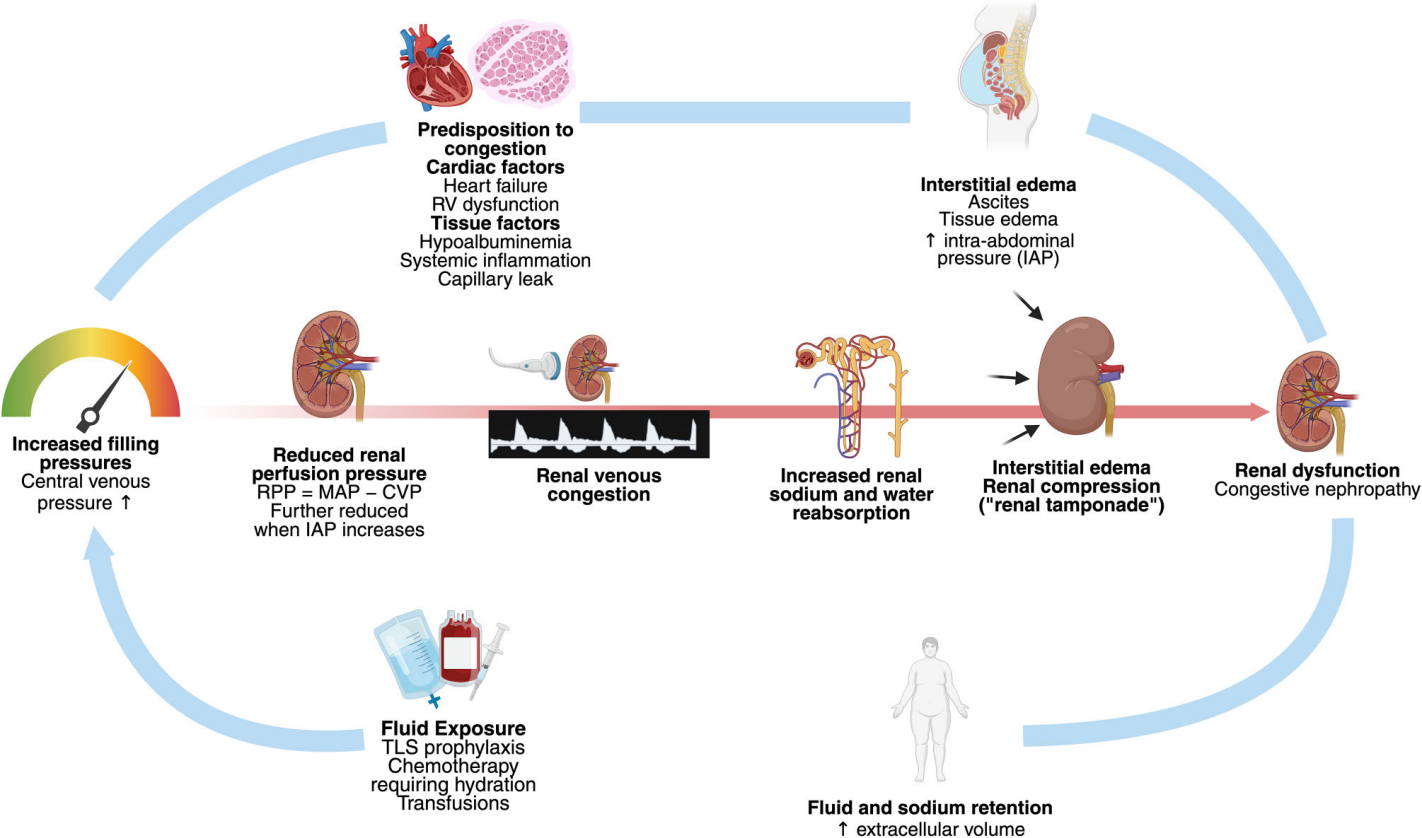
Pathophysiological cycle linking fluid exposure, VC, and congestive nephropathy in patients with cancer. Increased CVP reduces renal perfusion pressure (RPP = MAP − CVP), promoting renal VC, sodium retention, and interstitial edema, ultimately leading to kidney dysfunction and fluid retention. MAP, mean arterial pressure; RPP, renal perfusion pressure.

The pathophysiological link between FO and AKI is best explained by congestive nephropathy, a form of kidney dysfunction driven primarily by VC rather than arterial hypoperfusion (Fig. 1). In this condition, elevations in central venous pressure (CVP) are transmitted backward to the renal venous system, impairing venous outflow, increasing interstitial pressure, and reducing the effective transglomerular filtration gradient. Experimental and clinical studies consistently show that sustained increases in CVP can cause abrupt declines in glomerular filtration rate (GFR), even when systemic arterial pressure remains preserved [5, 18, 19].

VC may also promote sodium and water retention and further impair renal perfusion through increases in interstitial pressure and IAP [6, 20, 21]. An important extension of congestive nephropathy is the role of ascites and increased IAP. In cancer patients, ascites and abdominal FO are common due to advanced disease, peritoneal involvement, capillary leak, or aggressive fluid administration. As IAP rises, it compresses the kidneys, renal veins, and inferior vena cava, further impairing venous outflow and arterial inflow. When IAP approaches or exceeds CVP, effective renal perfusion pressure may collapse, leading to marked reductions in renal blood flow and GFR despite preserved systemic blood pressure [5, 6, 22, 23].

These mechanisms are frequently encountered in oncology and hematology, particularly in patients with ascites, abdominal distension, capillary leak, or aggressive fluid exposure. In such settings, additional fluid administration may worsen VC and IAP without improving renal perfusion, whereas de-resuscitation strategies or paracentesis may improve kidney function. Fluid accumulation is also common in advanced malignancies and has been associated with reduced survival and poorer functional status [6, 22–24].

Cancer is associated with an increased risk of heart failure across multiple tumor types, with population-based studies showing higher incidence after cancer diagnosis [25]. Importantly, cardiac filling pressures, the key determinant of congestion, are rarely directly measured in cancer patients. Current diagnostic approaches rely on surrogate markers such as left ventricular ejection fraction (LVEF), natriuretic peptides, and noninvasive estimates of filling pressures, while invasive hemodynamic assessment is limited to select cases [26].

This limitation is clinically relevant because symptoms such as dyspnea, edema, and reduced functional capacity are common in oncology and often multifactorial, making it difficult to distinguish true hemodynamic congestion from alternative mechanisms. Moreover, FO in cancer is mechanistically heterogeneous. Similar clinical presentations may result from VC, capillary leak syndrome, hypoalbuminemia, or treatment-related sodium retention, each requiring different therapeutic approaches.

Notably, tissue congestion can coexist with intravascular volume depletion in inflammatory states, where impaired lymphatic drainage and altered interstitial pressures promote fluid sequestration [27]. In such contexts, fluid administration may worsen edema without improving organ perfusion, exposing the limitations of uniform, protocol-driven fluid strategies and the need for individualized physiological assessment.

Taken together, evidence from onco-nephrology, population-based cohorts, critical care, and mechanistic studies converges on a consistent conclusion: FO is common and strongly associated with adverse outcomes in cancer patients. AKI in this population is often not solely due to intrinsic tubular injury or nephrotoxicity but reflects a potentially reversible hemodynamic and interstitial process. Recognizing FO and VC as upstream drivers of AKI provides a physiological basis for rethinking fluid management strategies in oncology and hematology. It also underscores the importance of bedside tools capable of assessing FT rather than relying solely on volume metrics.

### Limitations of conventional metrics and the potential of point-of-care ultrasound in the assessment of AKI

Serum creatinine, urine output, urinary biochemical indices, and physical examination are the standard tools used for AKI assessment; however, they often provide fragmented and potentially misleading information, particularly in oncology and hematology patients where FO and congestion are frequent.

Serum creatinine is a delayed and indirect marker of kidney function. In patients with cancer, reduced muscle mass due to sarcopenia, cachexia, malnutrition, and advanced age commonly leads to deceptively low baseline creatinine levels, resulting in overestimation of kidney function and delayed detection of AKI. Moreover, creatinine rises only after substantial reductions in GFR, and its interpretation is further confounded by hemodilution in patients receiving aggressive intravenous hydration. Consequently, clinically significant kidney stress may be present despite stable or even decreased creatinine levels [28, 29]. Creatinine changes should always be interpreted within clinical context, as transient increases may occur during effective decongestion despite improved hemodynamics and outcomes, whereas persistent congestion is associated with worse prognosis [30, 31].

Urine output is frequently used as a dynamic marker of kidney function and volume status, but its interpretation is also highly context-dependent. Oliguria may indicate reduced GFR but is often influenced by neurohormonal activation, VC, increased IAP, pain, stress, or vasopressor therapy. Conversely, transient increases in urine output following fluid administration may create false reassurance despite ongoing kidney stress [7, 32]. Urinary biochemical indices, particularly the fractional excretion of sodium (FENa) and urea (FEUrea), are commonly used to support the diagnosis of “prerenal” AKI [33, 34]. However, their reliability is limited in oncology patients, as they assume intact tubular function and stable neurohormonal conditions. In reality, systemic inflammation, sepsis, corticosteroid use, chemotherapy-induced tubular dysfunction, CKD, and diuretics significantly alter sodium and urea handling [35]. As a result, low FENa or FEUrea values may occur in congestion-mediated AKI and be misinterpreted as hypovolemia, potentially leading to inappropriate fluid administration [36, 37]. Thus, urinary indices are also context-dependent and should be interpreted cautiously.

Physical examination performs poorly in detecting congestion and estimating cardiac filling pressures. Classical signs such as peripheral edema, pulmonary crackles, and jugular venous distension have limited sensitivity and may be absent despite significant elevations in filling pressures. This limitation is especially problematic in oncology patients, where anemia, hypoalbuminemia, cachexia, obesity, pleural effusions, and treatment-related factors further confound clinical assessment [38, 39].

Given these limitations, conventional tools cannot reliably assess real-time hemodynamics, estimate filling pressures, or determine FT. In this context, PoCUS has emerged as a valuable extension of the physical examination, enhancing bedside assessment by providing real-time physiological information. PoCUS integrates focused cardiac ultrasound, lung ultrasound, and venous Doppler assessment, enabling evaluation of cardiac function, stroke volume, filling pressures, and systemic VC [40]. It has been conceptualized as a “fifth pillar” of bedside assessment, complementing traditional clinical tools [13–15].

**Figure 2:**
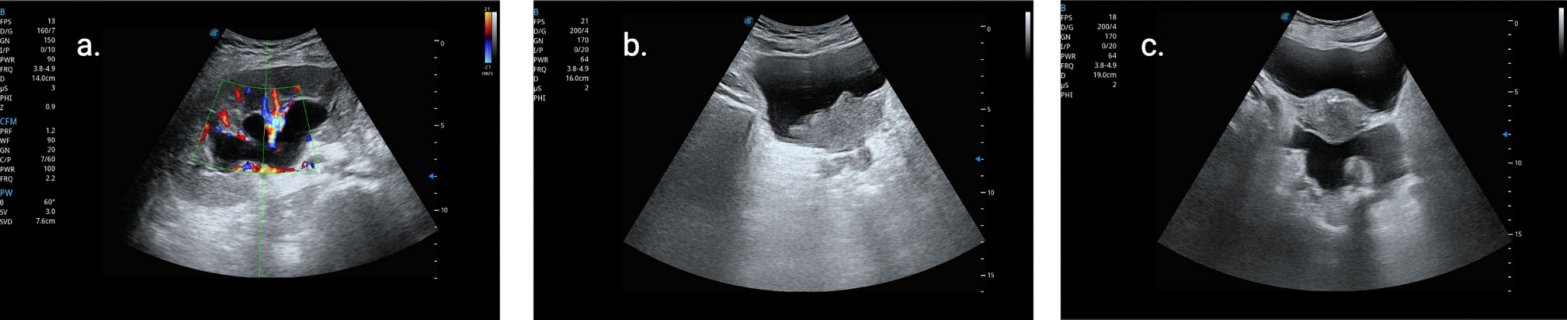
Representative PoCUS findings in oncology patients. (a) Hydronephrosis with dilation of the renal pelvis and calyceal system, consistent with obstructive uropathy. (b) Bladder ultrasound demonstrating an intraluminal mass arising from the bladder wall, suggestive of neoplastic involvement. (c) Pelvic ultrasound showing the urinary bladder (superiorly), the uterus (centrally), and surrounding ascitic fluid. These findings illustrate the value of PoCUS as a rapid bedside tool for identifying reversible postrenal causes of AKI and associated abdominal pathology in patients with cancer, facilitating timely diagnostic and therapeutic interventions. Images are original and were obtained by the authors.

In AKI evaluation, PoCUS first facilitates rapid identification of reversible postrenal causes. Renal and bladder ultrasound can detect hydronephrosis or bladder distension (Fig. 2), findings of particular importance in oncology where obstruction may result from tumors, lymphadenopathy, clots, retroperitoneal disease, or treatment-related urinary retention [41, 42]. Beyond obstruction, lung ultrasound provides a sensitive assessment of left-sided congestion. The presence of diffuse bilateral B-lines reflects increased extravascular lung water and correlates with elevated left-sided filling pressures. Notably, lung ultrasound often detects pulmonary congestion earlier than physical examination or chest radiography and can identify patients who are already fluid intolerant despite minimal symptoms [43–45]. However, pulmonary findings alone do not capture systemic VC, which is a major driver of AKI in many cancer patients.

To address this limitation, the Venous Excess Ultrasound Score (VExUS) has been developed [46]. Rather than estimating intravascular volume, VExUS evaluates the downstream effects of elevated right atrial pressure on the venous system. By combining inferior vena cava assessment with Doppler analysis of abdominal veins, VExUS provides a structured approach to identifying clinically significant VC affecting end organs, including the kidneys [47–49]. Interpretation of individual venous signals requires caution, particularly for intrarenal venous Doppler, which is technically demanding and context-dependent [50]. In contrast, portal vein Doppler is more robust, as increased pulsatility occurs only when hepatic buffering is overwhelmed. Elevated portal vein pulsatility is therefore a specific marker of significant systemic congestion and has been consistently associated with AKI and adverse outcomes [50, 51]. When abdominal windows are limited, alternative sites such as the internal jugular and femoral veins can be used. A plethoric internal jugular vein with reduced respiratory variation or a pulsatile femoral venous pattern indicates transmission of elevated right atrial pressure and systemic congestion [52, 53]. These concepts are especially applicable in oncology and hematology, where VC frequently develops in the setting of protocol-driven hydration, transfusions, capillary leak syndromes, corticosteroid exposure, ascites, or right-sided cardiac dysfunction. Nonetheless, direct validation studies of VExUS in cancer populations remain limited, and current applications are largely extrapolated from critical care and cardiorenal medicine.

Cardiac PoCUS provides essential upstream information to contextualize congestion. While qualitative assessment of left ventricular systolic function is useful, it is insufficient alone to guide fluid management. In this context, cardiac PoCUS allows estimation of stroke volume through measurement of the left ventricular outflow tract velocity–time integral (LVOT VTI) [28, 54]. LVOT VTI serves as a practical surrogate of CO. An illustrative case report from our group described a patient with multiple myeloma, high-output heart failure, systemic VC, and persistent AKI in whom multiorgan PoCUS demonstrated markedly elevated CO despite worsening kidney function. This case demonstrates that congestion-mediated kidney dysfunction may occur even in the presence of high forward flow and illustrates the value of PoCUS for identifying hemodynamic phenotypes that may not be apparent using conventional clinical assessment alone. Nevertheless, evidence supporting the use of cardiac PoCUS in onco-hematology remains largely extrapolated from heart failure and critical care populations and warrants further validation in cancer-specific settings [55].

Conversely, a low LVOT VTI suggests reduced forward flow and identifies patients who may benefit from cautious fluid administration or hemodynamic optimization [11, 56]. Emerging data also indicate that artificial intelligence-assisted handheld ultrasound devices can provide accurate and reproducible assessment of LVEF, even when used by nonexpert oncology clinicians, facilitating broader implementation in routine care [57].

**Figure 3:**
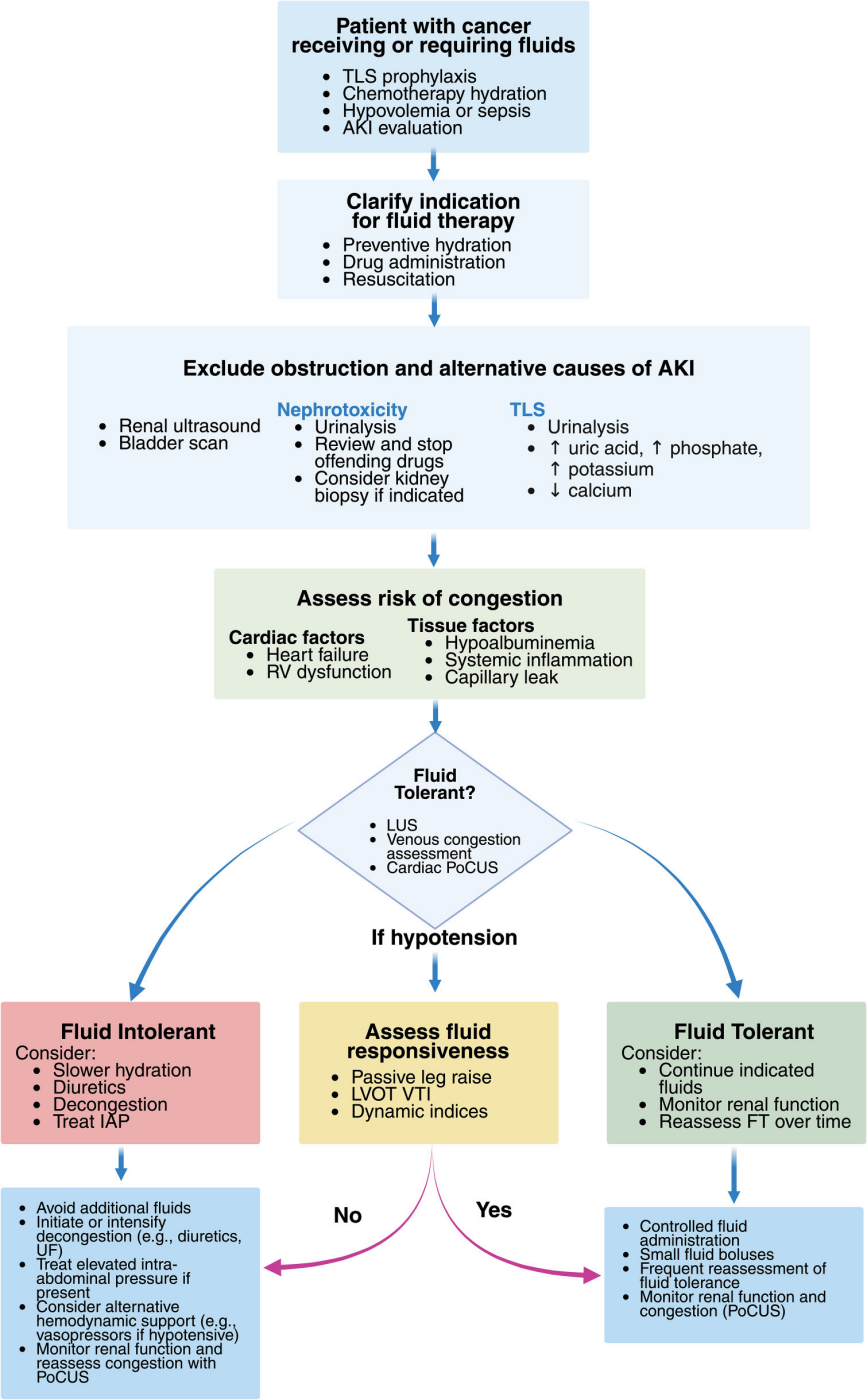
Physiology-guided approach to fluid management in patients with cancer receiving or requiring fluid therapy. LUS, lung ultrasound; UF, ultrafiltration.

By integrating lung ultrasound, VExUS, and cardiac PoCUS, clinicians can characterize congestion as left-sided, right-sided, or mixed, and differentiate between impaired forward flow and congestion-driven AKI. This integrated ultrasound approach allows clinicians to move beyond a one-size-fits-all fluid strategy, tailoring management to both FR and, critically, FT (Fig. 3).

Despite its advantages, several barriers limit widespread implementation of PoCUS in oncology and hematology. Effective use requires adequate training in image acquisition, interpretation, and clinical integration [58]. Variability in operator skill and interobserver agreement may affect reproducibility, particularly for advanced techniques such as venous Doppler and LVOT VTI measurement. Additionally, access to ultrasound equipment and trained personnel varies across institutions and healthcare systems [58, 59]. These challenges confirm that PoCUS is a skill-dependent modality requiring structured training and competency assessment. Nonetheless, it represents a promising tool to improve bedside evaluation and guide fluid management in patients with cancer and AKI.

### Fluid tolerance and fluid responsiveness: why “more fluids” is rarely a universal answer in onco-hematology

In critically ill patients, intravenous fluid administration has traditionally been approached as a binary decision, where hypovolemia prompts fluid administration and AKI is often interpreted as an indication for volume expansion. However, contemporary cardiovascular and renal physiology challenges this simplified view and reframes the clinical question into two key considerations: whether the patient will respond to fluids and whether the patient can tolerate them.

FR refers to the likelihood that fluid administration will increase CO, typically defined as a >10% increase in stroke volume following a preload challenge such as passive leg raise. FT, in contrast, refers to the capacity to receive fluids without developing clinically significant congestion or organ dysfunction, including AKI driven by elevated venous pressures rather than impaired circulating volume. Although related, FR and FT are distinct and dynamic properties that evolve over time [4]. These concepts carry particular weight in oncology and hematology, where patients are frequently exposed to fluids through hydration protocols, transfusions, contrast administration, sepsis management, and TLS prophylaxis. Despite this, “renal protection” is often still equated with liberal fluid administration.

FT in onco-hematology is highly context-dependent and often reduced in specific populations. Patients undergoing HSCT frequently experience endothelial injury, capillary leak, and sinusoidal obstruction syndrome, all of which predispose to FO [60]. Similarly, chimeric antigen receptor T-cell therapy is associated with cytokine release syndrome, characterized by systemic inflammation and capillary leak, increasing the risk of FO and VC [61, 62]. Patients with advanced multiple myeloma often present with renal impairment, hypoalbuminemia, and high disease burden, further limiting FT[63]. In these contexts, balancing the benefits and risks of fluid administration becomes particularly complex, making individualized FT assessment essential.

TLS represents a paradigmatic scenario in which hydration remains a cornerstone of prevention because it helps maintain renal perfusion and promotes urinary excretion of uric acid, phosphate, and potassium. Current guidelines consistently recommend intravenous hydration in patients at risk of TLS [10] (Table 1). Yet, although the physiological rationale is well established, the optimal volume and intensity of hydration remain uncertain, particularly in patients with limited FT [10]. Thus, our argument is not that hydration lacks physiological rationale, but that standardized hydration volumes may exceed FT in selected patients, particularly those with cardiorenal disease, CKD, systemic inflammation, or evolving AKI. Although aggressive hydration remains a cornerstone of TLS prophylaxis and is recommended in international guidelines, there is limited high-quality evidence supporting its efficacy in reducing AKI or improving outcomes, as no randomized trials have directly addressed this issue [8, 10, 64]. From a clinical perspective, the risk of FO is not uniform. Patients without significant comorbidities may tolerate higher fluid volumes, whereas those with pre-existing cardiorenal disease, advanced age, or systemic inflammation are at increased risk of congestion. Risk stratification is therefore central and based on tumor type, disease burden, proliferative rate, renal function, and treatment intensity. High-risk patients include those with acute leukemias, high-grade lymphomas, bulky disease, elevated lactate dehydrogenase, and baseline renal dysfunction [8, 10], highlighting that hydration protocols should be adapted to each patient's risk profile rather than applied uniformly.

Evidence from critically ill populations highlights the limitations of assuming that fluids are universally beneficial. In a multicenter study of 90 mechanically ventilated ICU patients with circulatory dysfunction, only 38% were fluid responsive, while 62% were not. Of note, VC was common even among fluid-responsive patients: 53% had at least one congestion marker, and 15% had multiple markers [65]. This identifies a subgroup in which CO may increase transiently despite underlying congestion, increasing the risk of organ injury. Indeed, the combination of FR and VC was independently associated with a higher risk of AKI at day 7 (odds ratio 4.33, 95% confidence interval 1.21–17.4) [65]. Conversely, absence of congestion does not imply FR; 43% of patients with normal venous ultrasound findings were fluid unresponsive [56]. When VExUS was used alone, specificity for identifying FR was only 13% [56]. These observations confirm that FR and FT provide complementary information and should not be used interchangeably.

Both FR and FT are dynamic and evolve over time. Babroudi *et al*. describe these as time-varying phenotypes, with patients transitioning within hours from responsive and tolerant to unresponsive and/or intolerant states [4]. This is supported by analyses from the ANDROMEDA-SHOCK trial, where only 13 patients remained fluid responsive throughout an 8-hour resuscitation period [66]. These findings illustrate how quickly the hemodynamic benefits of fluids can diminish during treatment.

These insights have direct implications for oncology and hematology practice, where fluid strategies often assume both FR and FT without formal assessment. The relevance of FR depends on the clinical context. In patients with hypotension, shock, or hypoperfusion, assessing FR is appropriate because increasing CO is a key therapeutic goal. By contrast, in normotensive patients with AKI, especially in preventive contexts such as TLS or chemotherapy hydration, the key considerations are the presence of obstruction and the patient’s ability to tolerate fluids. In these scenarios, the main risk is fluid intolerance and congestion rather than insufficient forward flow, and routine FR assessment may lead to unnecessary fluid administration.

TLS exemplifies this tension. Preventive hydration is typically initiated 24–48 hours before therapy at doses of 1–3 l/m^2^/day, aiming for urine output of ∼2 ml/kg/hour during the first 24–72 hours. Despite being considered a “fluid-friendly” scenario, aggressive hydration can lead to significant FO. In pediatric hematologic malignancies with TLS, severe FO has been reported in over one-third of cases and was associated with hypoxemia, pulmonary edema, and increased ICU needs [67]. Moreover, there is a lack of high-quality evidence defining optimal hydration volumes [10]. Guidelines and expert consensus documents recommend comparable high-volume hydration targets but emphasize close monitoring and caution in high-risk patients, acknowledging that even guideline-based hydration may exceed FT. Excessive hydration has been associated with pulmonary edema, ICU admission, need for RRT, and increased mortality in adults with AML [10, 17].

Taken together, these findings support a physiology-based approach to AKI in oncology and hematology. Evaluation should begin with exclusion of postrenal obstruction, a common and often overlooked cause related to tumors, lymphadenopathy, or treatment effects. Once obstruction is excluded, focus should shift to assessing FT, using tools such as PoCUS to evaluate VC and cardiac filling pressures. The frequency of repeat PoCUS assessments should be individualized according to the patient’s clinical status, severity of congestion, and response to therapeutic interventions, as no evidence-based monitoring interval has been established in oncology or hematology populations. FR assessment should be reserved for patients with hypotension or signs of low perfusion, using dynamic measures such as LVOT VTI. This integrated approach prioritizes mechanism-based management over reflexive fluid administration. In patients with cancer and AKI, it reinforces a key principle: fluids are a powerful therapy, but without careful consideration of tolerance, timing, and physiology, more fluids are rarely the right answer.

## CONCLUSIONS

AKI in oncology and hematology is often multifactorial, but congestion-related kidney dysfunction remains underrecognized and may be exacerbated by protocol-driven fluid administration. Conventional tools such as serum creatinine, urine output, and urinary indices provide limited information on FT and VC. PoCUS offers a practical bedside approach to assess congestion, identify patients at risk of FO, and guide individualized fluid management. Integrating physiology-based assessment into routine practice may help shift AKI management away from reflexive fluid administration toward more personalized and potentially safer fluid strategies. Further studies are needed to validate PoCUS-guided approaches and determine their impact on clinical outcomes in oncology and hematology populations.

## Data Availability

The data underlying this article are available in the article.
